# Whole-genome sequence of the filamentous diazotrophic cyanobacterium *Tolypothrix* sp. PCC 7712 and its comparison with non-diazotrophic *Tolypothrix* sp. PCC 7601

**DOI:** 10.3389/fmicb.2022.1042437

**Published:** 2022-11-08

**Authors:** Mahir Bozan, Denny Popp, Rene Kallies, Ulisses Nunes da Rocha, Stephan Klähn, Katja Bühler

**Affiliations:** ^1^Department of Solar Materials, Helmholtz-Centre for Environmental Research (UFZ), Leipzig, Germany; ^2^Department of Environmental Microbiology, Helmholtz-Centre for Environmental Research (UFZ), Leipzig, Germany

**Keywords:** next-generation sequencing, comparative genomics, cyanobacteria, *Tolypothrix*, *Fremyella diplosiphon*

## Abstract

Cyanobacteria are highly promising microorganisms in forthcoming biotechnologies. Besides the systematic development of molecular tools for genetic engineering, the design of chassis strains and novel reactor concepts are in focus. The latter includes capillary biofilm reactors (CBR), which offer a high surface area-to-volume ratio and very high cell densities. In this context, *Tolypothrix* sp. PCC 7712 was found to be highly suited for this reactor system due to maximal surface coverage, extraordinarily strong biofilm attachment, and high biomass formation. Here, we provide the genome sequence of *Tolypothrix* sp. PCC 7712 to potentially allow targeted strain engineering. Surprisingly, it was almost identical to an available incomplete genome draft of *Tolypothrix* sp. PCC 7601. Thus, we completely sequenced this strain as well and compared it in detail to strain PCC 7712. Comparative genome analysis revealed 257 and 80 unique protein-coding sequences for strains PCC 7601 and PCC 7712, respectively. Clustering genomes based on average nucleotide identity (ANI) and 16S rRNA homology showed 99.98% similarity and only minor distance, respectively, between the two strains in contrast to 21 other cyanobacterial genomes. Despite these high similarities, both strains differ in the ability to fix atmospheric nitrogen and show specific sequence variations, which are discussed in the paper.

## Introduction

Cyanobacteria are the only prokaryotes performing oxygenic photosynthesis; i.e., oxygen is released as a side product of light-driven water oxidation. The obtained electrons are used to drive an autotrophic metabolism based on CO_2_ fixation. Furthermore, multiple species are capable of fixing dinitrogen gas (N_2_; Tsygankov, 2007; Bharti et al., 2017). These features form the basis for a sustainable biotech-workhorse being independent of organic carbon and reduced nitrogen compounds, which usually add significantly to the ecological footprint of biotech processes. Although numerous proof-of-concept studies show the feasibility of using cyanobacteria as solar cell factories for producing commodity products (Angermayr et al., 2015; Betterle and Melis, 2019; Hoschek et al., 2019; Xie and Lindblad, 2022), only a few examples exist where cyanobacterial biocatalysts are applied at an economic scale. All these processes are based on biomass, which needs to be harvested to extract, e.g., pigments or lipids (Jones and Mayfield, 2012; Garlapati et al., 2019; Nagappan et al., 2020). Persisting challenges for applying cyanobacteria in production processes are low productivity and reaction stability, low cell densities due to light limitation, and insufficient light energy for efficient product conversion (Posten, 2009).

Recently, a novel reactor concept has been introduced, enabling long-term and high cell-density cultivation of cyanobacteria and potentially allowing for continuous production processes. In this regard, the unicellular model strain *Synechocystis* sp. PCC 6803 was grown as a biofilm in a capillary biofilm reactor (CBR; Hoschek et al., 2019). Biofilms are surface-attached microbial communities, supported and protected by a self-produced extracellular matrix containing mainly polysaccharides and other biopolymers like proteins, DNA, or lipids. They are widespread in nature with cyanobacteria playing a key role as primary producers in complex biofilms also termed microbial mats. In a biotechnological context, biofilms can be regarded as a robust biocatalyst, naturally immobilized to a given surface, enabling continuous bioprocessing (Halan et al., 2012).

In addition to *Synechocystis*, other cyanobacteria were screened for their utilization in CBRs (Bozan et al., 2022). In this survey, the filamentous, diazotrophic strain *Tolypothrix* sp. PCC 7712 (also known as *Gloeotrichia* sp.), first isolated from a soil sample collected in New York, United States, was identified as a top-performing organism. It was superior to all other strains investigated, e.g. in biofilm biomass formation, a low biofilm detachment rate, and high surface coverage. As processes that utilize cyanobacteria as solar cell factories aim to maximize biomass and maintain the cells in the reactor system, the above-mentioned parameters are important properties of an effective and productive catalytic biofilm. Nevertheless, to enable targeted engineering, including genetic modification of this promising cyanobacterium and to develop it further to become an established chassis strain, the genome sequence of *Tolypothrix* sp. PCC 7712 is required.

In order to establish this organism as a photo-biotech workhorse, we set out to sequence and analyze the genome of *Tolypothrix* sp. PCC 7712. Surprisingly, its genome sequence showed a high similarity to the available genome sequence of *Tolypothrix* sp. PCC 7601, also known as *Fremyella diplosiphon*, which was originally isolated from a freshwater sample at another location. Here, we present a comparative genome analysis of these two strains, which present distinct deviations in gene composition and arrangement causing substantial physiological differences between both strains. As for *Tolypothrix* sp. PCC 7601 only a permanent draft genome was available, we also provided a completed genome sequence for this strain.

## Materials and methods

### Cultivation and maintenance of strains

*Tolypothrix* sp. PCC 7712 and *Tolypothrix* sp. PCC 7601 were obtained from Pasteur Culture Collection of Cyanobacteria (PCC). Both strains were maintained on an agar-solidified BG11 medium (Rippka et al., 1979) in growth chambers (INFORS) at 25 μE m^−2^ s^−1^ illumination at 30°C. For the experiments, they were transferred to liquid media, either to standard BG11 or BG11-0 (nitrate omitted) in 250 ml flasks with 20 ml of culture volume. They were incubated at constant illumination of 25 μE m^−2^ s^−1^ at 30°C without shaking.

For determining the ability of chromatic light adaptation, bacterial pre-cultures growing in BG11-0 or BG11 media were transferred to fresh media after 3 weeks, covered with gray, red, and blue foil, and cultivated for another 3 weeks under 200 μE cm^−2^ s^−1^ light-emitting diode (LED) illumination. Whole-cell absorption spectra were analyzed using a Cary 300 UV–Vis spectrophotometer (Agilent Technologies, Santa Clara, United States).

### DNA isolation and quality assessment

Genomic DNA (gDNA) was isolated from *Tolypothrix* sp. PCC 7601 and PCC 7712 cells using established extraction protocols (Wilson, 2001) with few modifications. Briefly, 1 ml cell culture grown for 2 weeks was centrifuged at 13,000 *g* for 5 min, and the pellet was resuspended in 467 μl TE buffer. After addition of 100 μl Lysozyme (10 mg/ml), the pellet was resuspended by incubating it in a thermomixer (37°C, 15 min, 500 rpm), followed by the addition of 30 μl 10% SDS. The sample was incubated at the same conditions for another 15 min. Subsequently, 10 μl proteinase K (20 mg/ml) and 6 μl RNase (10 mg/ml) were added and the final solution was incubated for 1 h at 37°C while 400 rpm shaking. Then, pre-heated 80 μl 10% cetyltrimethylammonium bromide (CTAB) in 5 M NaCl solution was added together with 100 μl of pre-heated 5 M NaCl solution and incubated for 20 min at 65°C in a water bath. An equal volume of commercially obtained phenol/chloroform/isoamyl alcohol (25:24:1) was added to the mixture before it was centrifuged at 13,000 *g* for 10 min. The upper phase was transferred to a new tube followed by the addition of an equal volume of chloroform/isoamyl alcohol (24:1). After centrifugation (13,000 *g*, 10 min), the upper phase was transferred to a new tube, and 0.7 volume of isopropanol was added to the mixture. DNA was precipitated in isopropanol solution with centrifugation (13,000 *g*, 15 min). The pellet was washed with 70% ethanol. After removing the ethanol from the mixture by centrifugation, it was left open at 37°C for 1 h in order to remove residual ethanol from the gDNA pellet. The final pellet was resuspended with ddH_2_O and stored at 4°C. The quality and quantity of gDNA were checked *via* Nanodrop One^C^ Spectrophotometer (Thermo Fisher Scientific, Waltham, United States) at 260 and 280 nm.

### Whole-genome sequencing, assembly, and annotation

For Illumina sequencing, isolated gDNA was fragmented and a sequencing library was prepared using the NEBNext® Ultra™ II DNA Library Prep Kit for Illumina® (New England Biolabs) according to the manufacturer’s instructions. Sequencing was performed on an Illumina MiSeq platform with a MiSeq Reagent Kit v3 (600-cycle; Illumina). Adapter sequences from Illumina raw sequencing data (PRJNA625426 for PCC7712 and PRJNA625641 for PCC7601) were trimmed using BBDuk of the bbmap suite v38.33.^1^ In addition, whole genome sequencing was performed by PCR-free Nanopore sequencing using an R9.4.1 flow cell on a MinIon MK1B device (Oxford Nanopore Technologies) controlled by MinKnow software release 19.12.5. gDNA isolates were prepared for sequencing using an SQK-LSK 109 Ligation Sequencing Kit in combination with an EXP-NBD104 Native Barcoding Expansion Kit according to the manufacturer’s instructions with the following exceptions. The incubation times for the end-repair step were increased to 30 min at room temperature and 30 min at 65°C. The time for the ligation step was increased to 60 min. Raw Nanopore sequence data were base called and demultiplexed using guppy version 3.6.0 and the provided high accuracy model. Adapter sequences were trimmed using Porechop version 0.2.4.^2^ Genomes were assembled using (a) unicycler v0.4.8 in hybrid mode using Illumina and Nanopore reads (Wick et al., 2017) and (b) Flye v2.8 using Nanopore reads only (Kolmogorov et al., 2020). The resulting assemblies were polished by medaka v1.0.3 using the respective Nanopore reads^3^ and by four rounds of pilon v1.22 (Walker et al., 2014) using the respective Illumina reads. Final assembly quality was checked with CheckM v1.011 using the lineage-specific workflow (Parks et al., 2015) and quast v5.0.2 (Gurevich et al., 2013). After completing assemblies, final sequences were submitted to Genbank (PRJNA625426 for PCC7712 and PRJNA625641 for PCC7601) and annotated *via* PGAP pipeline.^4^ Plasmid types were identified with MOB-Recon (Robertson and Nash, 2018).

### Genome comparison

Both genomes were compared using Diffseq (Aggeli et al., 2018) to get information about genome variations between the two strains. MAUVE alignment (Darling et al., 2004) was applied to align chromosomes and plasmids to rearrange their initial locations for further Diffseq analyses. The genomes and generation of the annotation list were visualized using Geneious R10.0.5 (Kearse et al., 2012). To identify unique proteins BLAST RBH implemented to the Galaxy server^5^ was applied by comparing the encoding nucleotide sequences. After obtaining a “first match” list, respective sequences were extracted as a fasta file and BLAST RBH was used again against this extracted list for each genome. This process was repeated three times in total to avoid errors caused by multi-copy genes.

### Pairwise comparison of different genome sets

Twenty-one other cyanobacterial species listed in Supplementary Table S3, which are either well-known in the biotechnology field or compose other *Tolypothrix* species were selected from the NCBI genome database to compare them to the newly sequenced two genomes used in this study. After accessing the selected genomes, dRep (Olm et al., 2017) MASH ANI clustering was applied in the Galaxy Server https://usegalaxy.eu/. The primary clustering ANI threshold was set to 90% and the secondary clustering ANI threshold was set to 99%.

### Acetylene reduction assay

The acetylene reduction assay was applied as described (Stewart et al., 1968; Yoon and Golden, 2001) with some modifications to quantify nitrogenase activity *in vivo*. The respective cultures were adapted to nitrate-omitted media (BG11-0) for 1 week. Cells were harvested and transferred to 15 ml fresh BG11-0 medium in 20 ml GC vials; cell density was adjusted based on Chl*a* content to 1.5 μM Chl*a* which was measured according to the procedure described in a previous study (Zavřel et al., 2015). Briefly, a cell pellet obtained from 1 ml of cell culture was exposed to 1 ml of 100% methanol solution for 20 min in the dark followed by measuring the absorbance of the supernatant at 470, 665, and 720 nm wavelength *via* a visible range spectrophotometer (Libra S11, Biochrom, Cambridge, United Kingdom). After adjusting cell density *via* Chl*a* measurement, the 5 ml headspace was filled with acetylene (0.5%), oxygen (20.9%), and nitrogen (78.6%) gas mixture. Vials were left for incubation at 50 μE m^−2^ s^−1^ for 24 h before measuring ethylene production *via* gas chromatography (TRACE 1310; Thermo Scientific, Waltham, USA). The device was equipped with a 30 m long TracePLOT TG-BOND Q+ column with 0.32 mm inner diameter and 10 μm film thickness (Thermo Scientific, Waltham, USA). The temperature of the flame ionization detector and the oven were adjusted to 300 and 60°C, respectively. A volume of 10 μl was injected *via* a Thermo TriPlus RSH autosampler. The flow rate of the carrier gas (nitrogen) was set to 10 ml min^−1^, with a total running time of 2 min. The calibration curve was set by injecting 0.5, 1, and 2 μl of ethylene gas.

## Results

### Characterization and classification of the whole genome sequence of *Tolypothrix* sp. PCC 7712

The genome sequence of *Tolypothrix* sp. PCC 7712 was obtained by using a hybrid sequencing approach based on two different techniques, namely Illumina and Nanopore. The full genome sequence of *Tolypothrix* sp. PCC 7712 consisted of one large contig covering 9 Mbp and 15 smaller contigs of 0.03–0.2 Mbp in length (Table 1). The overall GC content was 40.7%.

**Table 1:** Summary of the *Tolypothrix* sp. PCC 7712 genome as obtained from Illumina and Nanopore sequencing.

Sequence name	Size (nt)	Genes	GC %	tRNA genes	Scaffold type
CP063785	9,007,860	7,133	40.8	78	Chromosome
CP063786	214,777	194	39.8	0	Conjugative
CP063787	192,899	158	39.7	0	Mobilizable
CP063788	176,706	152	39.2	24	Mobilizable
CP063789	92,234	72	41.1	0	Conjugative
CP063790	57,284	58	42.2	0	Mobilizable
CP063791	46,852	32	41.3	0	Non-mobilizable
CP063792	42,661	42	41.6	0	Mobilizable
CP063793	42,150	34	41.8	0	Mobilizable
CP063794	39,434	34	41.5	0	Mobilizable
CP063795	39,072	36	40.5	0	Mobilizable
CP063796	37,441	37	40.6	0	Mobilizable
CP063797	36,483	35	40.7	0	Non-mobilizable
CP063798	31,341	31	41.5	0	Mobilizable
CP063799	29,597	33	42.9	0	Mobilizable
CP063800	29,106	30	41.9	0	Mobilizable
Total	10,115,897	8,111	40.7	102	

Scaffold type was determined with MOB-Recon (Robertson and Nash, 2018). This module provided information about the type of the plasmid by matching them to plasmid reference databases and about plasmid transferability, replicon family, mate-pair formation, and relaxase type.

The largest contig (CP063785) of around 9 Mbp represents the chromosome, whereas several of the other contigs were predicted to be mobilizable and thus could represent plasmids. For the latter, we used the plasmid prediction tool MOB-Recon (Robertson and Nash, 2018). It should be noted that still several of these contigs might represent parts of the chromosome, especially when considering typically essential elements such as tRNAs. While the chromosome contig CP063785 contains 78 tRNA genes, 24 additional tRNA genes are found on contig CP063788, which possibly represents a plasmid. Nevertheless, plasmids that harbor tRNA arrays have also been reported for other bacterial species (Tran et al., 2016). Altogether, a set of 7,133 genes were identified on the largest contig and 978 genes, including 24 tRNA genes were found on the smaller contigs (Table 1).

**Figure 1:**
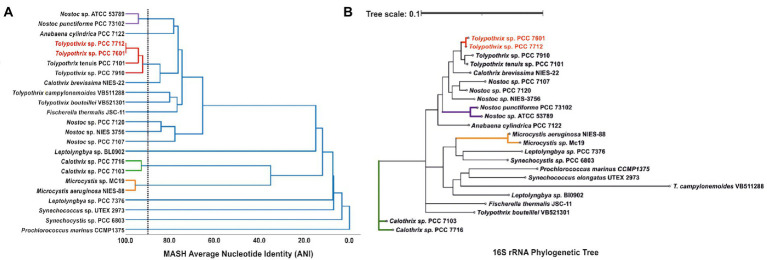
**(A)** The tree shows genome sequence similarity of 23 cyanobacterial strains including PCC 7601 and PCC 7712 as calculated by dRep MASH ANI clustering. The dotted line shows the primary ANI clustering threshold, which was set to 90%. **(B)** The phylogenetic tree indicates the distances between these strains based on 16S rRNA homology.

Random sequences from the obtained PCC 7712 genome were manually analyzed using BLASTN. Remarkably, they appeared to be identical to an available genome sequence, namely that of *Tolypothrix* sp. PCC 7601 even though both strains were isolated independently and at different geographical locations. To exclude possible cross-contaminations, both strains were reordered from the PCC, gDNA was isolated and the sequencing approach was performed again for both strains (resulting in the read and assembly statistics given in Supplementary Table S1). The average nucleotide identity (ANI) of both obtained genome sequences was determined using the method FastANI (Jain et al., 2018). Indeed, both strains showed 99.98% ANI, which makes the two strains closely related but also indicates particular sequence alterations. As expected from high ANI, dRep (Olm et al., 2017) MASH ANI clustering with 21 selected cyanobacterial genomes showed only minor distance between *Tolypothrix* sp. PCC 7601 and PCC 7712, which is also reflected by a phylogenetic tree based on 16S rRNA comparison (Figure 1). The most related strains based on ANI clustering and the 16S rRNA based phylogenetic tree were *Tolypothrix tenui*s PCC 7101 followed by *Tolypothrix* sp. PCC 7910.

**Figure 2:**
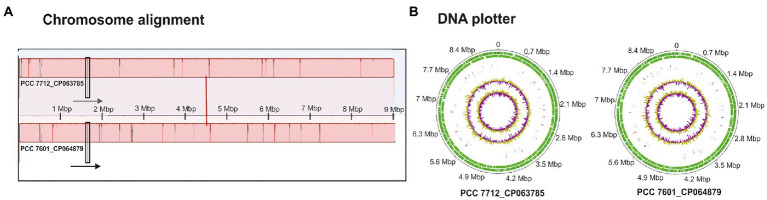
**(A)** Chromosome alignment together with the direction of sequences (arrows) and **(B)** DNA plotters of the two chromosomes of *Tolypothrix* sp. PCC 7712 and PCC 7601 showing GC and GC skew in the middle followed by RNAs (blue and red lines), and the outer line shows the sequence itself with its complementary strand (green).

Despite these high similarities of *Tolypothrix* sp. PCC 7712 and PCC 7601, one strain might have genes that the other does not have, or the order of genes might differ significantly. Therefore, gene order and genome synteny were examined using MAUVE to reveal potential differences. However, also in this respect both genomes showed high similarity in their genome arrangement (Figure 2).

### Comparative analysis of the genomes of *Tolypothrix* sp. PCC 7712 and PCC 7601 at global scale

*Tolypothrix* sp. PCC 7601 is the closest relative of strain PCC 7712. So far only an incomplete genome sequence of PCC 7601 containing 74 assembly gaps was available as a permanent draft deposited at GenBank (GCA_002368275.1). We, therefore, performed a hybrid-sequencing approach and achieved a complete circular chromosome of PCC 7601, as well as for strain PCC 7712 without any assembly gaps (Supplementary Figure S1), allowing for a detailed comparison of both strains using different bioinformatics tools.

**Figure 3:**
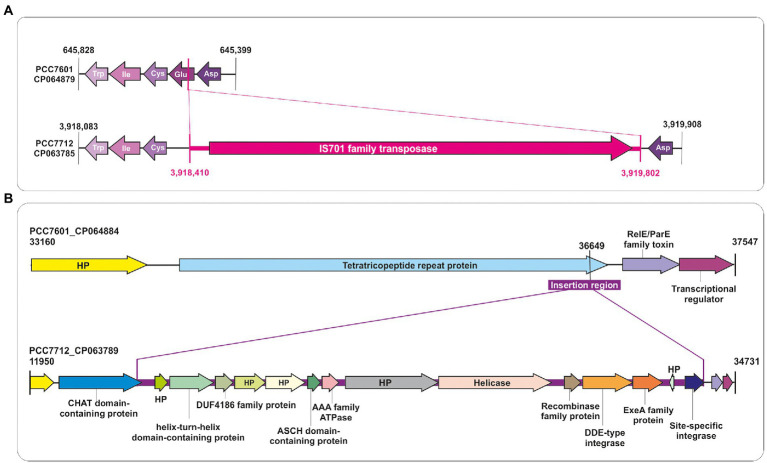
Comparative analysis of the genomes of *Tolypothrix* sp. PCC 7712 and PCC 7601 at global scale. **(A)** Insertion of a transposase belonging to the IS701 family located in the gene encoding the tRNA-Glu in the PCC 7712 chromosome. **(B)** Comparison of contigs PCC 7601_CP064884 and PCC 7712_CP063789, which differ by a large insertion sequence containing a number of protein-coding regions like five hypothetical proteins (HP), a helix-turn-helix domain-containing protein, an ASCH domain-containing protein, a DDE-type integrase, and an ExeA family protein.

To enable a detailed genome comparison of PCC 7712 and PCC 7601, including gene arrangement and composition, and identification of differences at gene as well as at nucleotide level, both strains’ genomes were annotated using the NCBI prokaryotic genome annotation pipeline (PGAP). This resulted in the annotation given in Supplementary Table S2. The annotation revealed that strain PCC 7712 lacks one tRNA, which was identified as a tRNA-Glu. This difference appeared to originate from the insertion of an IS701 family transposase (Figure 3A). Nevertheless, the number of encoded tRNAs already indicated that there is tRNA redundancy in *Tolypothrix* genomes, which also includes multiple tRNA-Glu with the same codon usage.

Moreover, a whole-genome comparison was carried out using Diffseq (Aggeli et al., 2018), which revealed differences between both genomes in gene composition as well as at the single nucleotide level. Both strains harbor several unique genes (Table 2). For instance, the possible plasmid CP063789 of strain PCC 7712 contains an insertion that significantly alters the gene composition compared to strain PCC 7601 (Figure 3B). It contains several unique genes such as for an activating signal cointegrator 1 homology (ASCH) domain-containing protein, a helix-turn-helix domain-containing protein, an AAA family ATPase, a helicase, a DUF4186 family protein, or an ExeA family protein and several hypothetical proteins. The region of insertion showed a high query coverage to proteins also found in closely related cyanobacterial strains of the genera *Anabaena*, *Nostoc* or *Calothrix*. ExeA family protein was reported to be involved in type II secretion systems (Vanderlinde et al., 2014), which might play a role in plasmid maintenance (Zhang et al., 2020). Further open reading frames (ORF) were uniquely identified either in the genome of strain PCC 7712 or PCC 7601 (see Table 2). Altogether, 80 unique CDS annotations were identified in strain PCC 7712 and 257 in strain PCC 7601. However, 199 CDS out of 257 (77%) in the genome of PCC 7601 and 41 out of 80 (51%) in the genome of PCC 7712 were identified as coding for hypothetical proteins (for a full list of unique ORFs see Supplementary Excel File S1).

**Table 2:** Examples of unique protein-coding regions found in the genome of PCC 7601 and PCC 7712.

*Tolypothrix* sp. PCC 7712			*Tolypothrix* sp. PCC 7601		
Sequence Name	**Locus_tag**	**Annotation**	**Sequence Name**	**Locus_tag**	**Annotation**
**CP063785**	HGR01_00210	CPXCG motif-containing cysteine-rich protein	**CP064893**	HG267_41510	3′-5′ Exonuclease
**CP063785**	HGR01_00215	SRPBCC family protein	**CP064879**	HG267_01625	Alpha/beta hydrolase
**CP063785**	HGR01_12620	Alpha/beta hydrolase	**CP064879**	HG267_09120	Carbamoylphosphate synthase large subunit
**CP063785**	HGR01_16155	CTB family bacteriocin	**CP064879**	HG267_25985	DDE-type integrase/transposase/recombinase
**CP063789**	HGR01_38235	Helix-turn-helix domain-containing protein	**CP064879**	HG267_07765	DUF3854 domain-containing protein
**CP063789**	HGR01_38255	ASCH domain-containing protein	**CP064879**	HG267_32890	Formylglycine-generating enzyme family protein
**CP063789**	HGR01_38260	AAA family ATPase	**CP064893**	HG267_41505	HAD hydrolase-like protein
**CP063789**	HGR01_38270	Helicase	**CP064879**	HG267_13395	Helicase
**CP063789**	HGR01_38285	ExeA family protein	**CP064879**	HG267_07695	Helix-turn-helix transcriptional regulator
**CP063789**	HGR01_38290	Hypothetical protein	**CP064879**	HG267_07440	Histidine kinase
**CP063789**	HGR01_38295	Site-specific integrase	**CP064879**	HG267_00005	Hypothetical protein
**CP063797**	HGR01_39915	EAL domain-containing protein	**CP064879**	HG267_04935	IS5 family transposase
**CP063797**	HGR01_39920	Restriction endonuclease	**CP064882**	HG267_38370	IS66 family transposase
**CP063797**	HGR01_39925	Tyrosine-type recombinase/integrase	**CP064879**	HG267_01590	IS701 family transposase
**CP063797**	HGR01_39935	GIY-YIG nuclease family protein	**CP064879**	HG267_12995	ltrA
**CP063797**	HGR01_39985	6-Aminohexanoate hydrolase	**CP064879**	HG267_32895	Mechanosensitive ion channel
**CP063797**	HGR01_40010	PT domain-containing protein	**CP064883**	HG267_39545	Relaxase/mobilization nuclease domain-containing protein
**CP063797**	HGR01_40025	Pentapeptide repeat-containing protein	**CP064879**	HG267_07790	Ribbon-helix–helix protein, CopG family
**CP063797**	HGR01_40030	Pentapeptide repeat-containing protein	**CP064893**	HG267_41535	Site-specific integrase
**CP063797**	HGR01_40065	AAA family ATPase	**CP064879**	HG267_25980	Tn7 transposase TnsA N-terminal domain-containing protein
**CP063797**	HGR01_40080	Helix-turn-helix transcriptional regulator	**CP064879**	HG267_00120	Transposase
**CP063797**	HGR01_40085	Integrase	**CP064879**	HG267_09560	Type II toxin-antitoxin system ParD family antitoxin
**CP063798**	HGR01_40105	DUF3854 domain-containing protein	**CP064879**	HG267_07845	Tyrosine-type recombinase/integrase
**CP063798**	HGR01_40165	DUF3883 domain-containing protein			
**CP063798**	HGR01_40170	Site-specific integrase			
**CP063798**	HGR01_40180	DNA cytosine methyltransferase			
**CP063798**	HGR01_40190	ATP-binding protein			
**CP063798**	HGR01_40195	Type II toxin-antitoxin system VapC family toxin			
**CP063798**	HGR01_40210	AAA family ATPase			
**CP063798**	HGR01_40215	S1 RNA-binding domain-containing protein			
**CP063798**	HGR01_40225	TIR domain-containing protein			

The list was initially obtained by BLAST RBH and then finalized after iterative blast searches with unmatched proteins subsequently. For a full list of unique ORFs, see Supplementary Excel File S1.

### Comparative analysis of both genomes at the single nucleotide level

Moreover, numerous single nucleotide polymorphisms (SNPs) were detected, mostly in the locations of mobile elements such as IS5, IS1634, and ISKra4 family transposases. Table 3 summarizes selected examples of the SNPs found in the genome of PCC 7712. However, most of the SNPs have been found in the non-coding regions of the genome rather than in the coding regions. These could become interesting if they affect *cis*- or *trans*- genetic elements, promotors or ribosomal binding sites. Some were also located within protein-coding regions, e.g., affecting an ATP/GTP-binding protein, HAMP domain-containing histidine kinase, and SDR family NAD(P)-dependent oxidoreductase all having a non-synonymous SNP, resulting in amino acid sequence alterations. In contrast, a gene for a tetratricopeptide repeat protein had synonymous SNP, not affecting the corresponding amino acid sequence.

**Table 3:** Examples of SNPs and insertions found in the genome of PCC 7712 compared to PCC 7601. For a complete list please refer to Supplementary Excel File S1.

Contig	Contig position (start)	Contig position (end)	Putative function	Type of variation
PCC7712_CP063785	50,753	52,296	Radical SAM protein CPXCG motif-containing cysteine-rich protein SRPBCC family protein	Insertion of 1,544 bases
PCC7712_ CP063785	231,980	233,896	ISKra4 family transposase Hypothetical protein	Insertion of 1917 bases
PCC7712_ CP063785	258,093	258,093	AAA-like domain-containing protein	Insertion of one base
PCC7712_ CP063785	286,946	287,017	Cobyrinate a,c-diamide synthase	Insertion of 72 bases
PCC7712_ CP063785	1,463,324	1,463,324	DEAD/DEAH box helicase	Insertion of one base
PCC7712_ CP063785	2,322,677	2,322,678	Helix-turn-helix domain-containing protein	Insertion of two bases
PCC7712_ CP063785	4,128,214	4,128,220	Alpha/beta hydrolase	Insertion of seven bases
PCC7712_ CP063785	6,314,548	6,314,554	Hypothetical protein	Insertion of seven bases
PCC7712_ CP063785	7,018,704	7,018,710	Iron ABC transporter permease	Insertion of seven bases
PCC7712_ CP063785	819,728	819,728	IS5 family transposase	SNP
PCC7712_ CP063785	1,218,062	1,218,062	IS1634 family transposase	SNP
PCC7712_ CP063785	1,537,608	1,537,608	ATP/GTP-binding protein	SNP
PCC7712_ CP063785	1,854,700	1,854,700	psbD	SNP
PCC7712_ CP063785	2,144,126	2,144,126	ISKra4 family transposase	SNP
PCC7712_ CP063785	2,411,161	2,411,161	23S rRNA	SNP
PCC7712_ CP063785	3,712,226	3,712,226	16S rRNA	SNP
PCC7712_ CP063785	3,996,355	3,996,355	Non-coding region of CTB family bacteriocin	SNP
PCC7712_ CP063785	3,996,451	3,996,451	CTB family bacteriocin	SNP
PCC7712_ CP063785	5,956,540	5,956,540	Tetratricopeptide repeat protein	SNP
PCC7712_ CP063785	7,391,583	7,391,583	SDR family NAD(P)-dependent oxidoreductase	SNP
PCC7712_ CP063785	8,035,613	8,035,613	HAMP domain-containing histidine kinase	SNP
PCC7712_ CP063786	23,958	23,991	DUF2127 domain-containing protein	Insertion of 34 bases
PCC7712_ CP063787	180,725	180,743	DUF1822 family protein	Insertion of 19 bases
PCC7712_ CP063788	138,690	140,082	IS701 family transposase, hypothetical protein	Insertion of 1,393 bases
PCC7712_ CP063788	62,134	62,134	Hypothetical protein	SNP
PCC7712_ CP063788	62,688	62,688	DUF1822 family protein	SNP
PCC7712_ CP063789	15,435	33,835	hypothetical protein(5x), helix-turn-helix domain-containing protein, DUF4186 family protein, ASCH domain-containing protein, AAA family ATPase, helicase, recombinase family protein, DDE-type integrase/transposase/recombinase, ExeA family protein, and site-specific integrase	Insertion of 18,401 bases
PCC7712_ CP063790	35,960	35,960	ltrA (group II intron reverse transcriptase/maturase)	Insertion of one base
PCC7712_ CP063790	45,404	45,404	TniQ family protein	Insertion of one base
PCC7712_ CP063799	27,712	27,728	ATP-binding protein	Insertion of 17 bases
PCC7712_ CP063799	21,725	21,725	ItrA (group II intron reverse transcriptase/maturase)	SNP
PCC7712_ CP063800	9,133	9,139	Protein kinase	Insertion of seven bases

### *Tolypothrix* strains PCC 7712 and PCC 7601 are closely related but differ in nitrogen fixation ability

**Figure 4:**
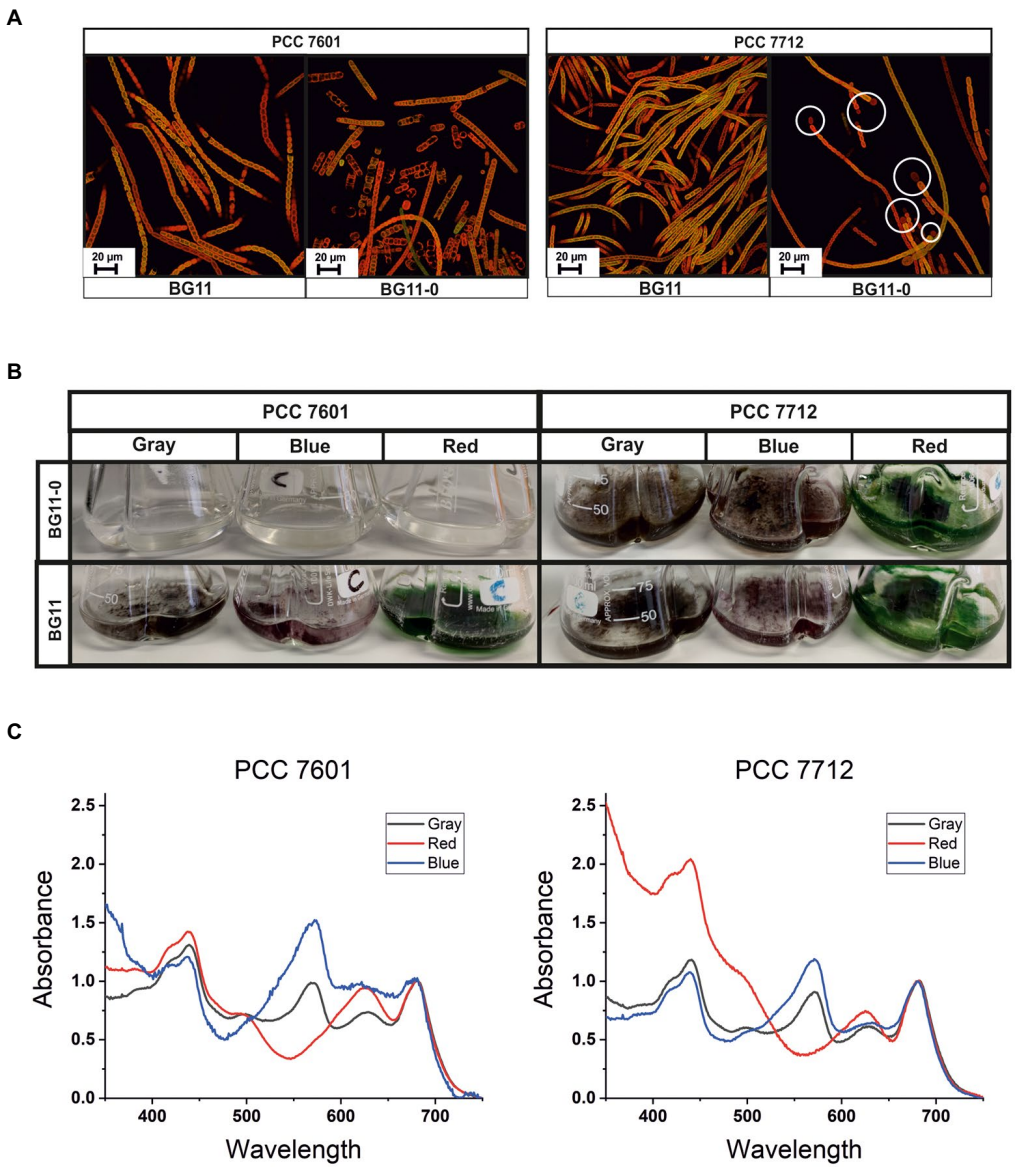
**(A)** CLSM images of PCC 7601 and PCC 7712 cultures growing in BG11-0 or BG11 media; white circles indicate heterocyst cells, which are less fluorescent as they lack pigmentation. **(B)** Cultures growing in different light spectra in shake flasks in either BG11-0 or BG11. **(C)** Chromatic light adaptation. Whole-cell absorption spectra of cultures covered with different colored foils—red, blue, and gray.

Microscopy analyses showed that both PCC 7712 and PCC 7601 have similar morphology (Figure 4A). Both are filamentous and the filaments are generally entangled in some regions, which explains their hairy flocs appearance in suspended batch cultures (Figures 4A,B). Furthermore, pigmentation and the ability of chromatic adaptation are fairly similar in both strains (Figures 4B,C), which is also indicated by the sequence identity in the respective proteins involved such as phytochrome superfamily photoreceptors RcaE and DpxA (Bordowitz and Montgomery, 2008; Wiltbank and Kehoe, 2016). *Tolypothrix* sp. PCC 7601 is a model organism for investigating the mechanism behind complementary chromatic acclimation (CCA; Grossman et al., 2001), while this phenomenon has not been described for PCC 7712 so far, but is obviously also existing in this strain.

A major physiological difference between the two strains is the ability of PCC 7712 to fix dinitrogen gas and thus being able to grow in a medium lacking an assimilable nitrogen source like nitrate (BG11-0). Under nitrate-limiting conditions only strain PCC 7712 formed terminal heterocysts (Figure 4A; white circles), which are specialized cells harboring the nitrogenase needed for N_2_ fixation. In contrast, strain PCC 7601 showed stressed filaments in BG11-0 lacking clear terminal heterocysts. Furthermore, no cell growth was observed which is consistent with previous reports (de Alda et al., 2004).

We performed an acetylene reduction assay to quantify the nitrogenase activity under aerobic conditions to confirm these observations. As expected, strain PCC 7601 did not show any activity, while a fairly high nitrogenase activity was detected in strain PCC 7712 converting up to 26.1 μmol H_2_ mg_Chl*a*_^−1^ h^−1^ under here used assay conditions. Strikingly, this major difference was not observable on the genomic level based on current state-of-the-art knowledge about nitrogen fixation in *Tolypothrix*. Even though there were several unique proteins, insertions, and SNPs observed in both genomes, none of the differences were identified in the regions currently known to be related to nitrogen fixation or heterocyst formation genes (Tables 2, 3).

## Discussion

*Tolypothrix* sp. PCC 7712 is a novel candidate strain for cyanobacterial photo-biotechnology research due to its high biofilm biomass formation, low detachment rate, and high surface coverage in a recently introduced CBR system (Bozan et al., 2022). On the way to establish this organism as a potential chassis strain, the genome of *Tolypothrix* sp. PCC 7712 was sequenced and analyzed. We used the ANI approach, which, in contrast to the 16S rRNA-based classification, considers the total genome, and is regarded as a much more reliable tool for deciphering the degrees of bacterial relation (Jain et al., 2018). Even though strain PCC 7712 originated from a different geographical region it showed 99.9871% sequence identity with strain PCC 7601. Therefore, one could assume that both strains would have a similar phenotype despite minor differences in their genomes. This, for instance, is the case for complementary chromatic acclimation (CCA). *Via* CCA, cyanobacteria are able to change the arrangement of proteins and pigments in their phycobilisomes as well as in the accessory light-harvesting complexes connected to their photosystems when exposed to an altered light quality such as red or green light. While strain PCC 7601 has been widely used as a model strain for understanding the mechanism behind CCA (Kehoe and Gutu, 2006; Gutu and Kehoe, 2012; Montgomery, 2016), this phenomenon was so far not described for strain PCC 7712.

The most striking physiological difference was the ability of PCC7712 to fix dinitrogen gas *via* terminal heterocysts, while PCC 7601 was relying on dissolved inorganic nitrogen compounds like nitrate. This came as a surprise, as the genome of PCC 7601 harbors all essential genes for nitrogen fixation and heterocyst formation with 100% sequence identity to PCC 7712. Besides differences listed in Tables 2, 3 and Supplementary Excel File S1, there are no other differences on nucleotide level, which allow a conclusion on the reason for this observation.

Interestingly, a spontaneous revertant, *Tolypothrix* sp. PCC 7601/1 was reported previously, which is able to form heterocysts and fix dinitrogen (de Alda et al., 2004). Unfortunately, there are no sequencing data available for this strain but likely SNPs play a role in this regard. Moreover, it has been shown previously that the phycobilisome degradation protein NblA1 might play a role in the heterocyst differentiation of *Tolypothrix* sp. PCC 7601/1 but the underlying mechanism is not clearly identified yet (de Alda et al., 2004). This protein was found highly abundant under nitrogen limiting conditions in PCC 7601/1; however, it has been shown that it is also present in nitrogen replete conditions. Therefore, it was hypothesized that differential expression of the *nblA1* gene could be involved in the complex heterocyst differentiation in filamentous cyanobacteria (de Alda et al., 2004). However, when comparing both PCC 7712 and PCC 7601, we could not determine any differences neither in the *nblA1* gene nor in respective up- and downstream sequences. Moreover, there are several reports on small non-coding RNAs that are important during heterocyst differentiation in *Nostoc* sp. PCC 7120 (Brenes-Álvarez et al., 2020, 2022). Yet, a whole-genome comparison of PCC 7712 and PCC 7601 did not show any differences for small non-coding RNAs at the genome level. Nevertheless, it might be worth investigating the transcriptomes of PCC 7712 and PCC 7601 under different conditions to reveal non-coding RNAs and to unravel further details about the complex heterocyst formation process together with nitrogen fixation. Furthermore, regulatory effects based on epigenetic DNA modifications should also be considered as we identified a DNA cytosine methyltransferase (HGR01_40180) as a unique protein-coding sequence in the genome of PCC 7712 (Table 2). The presence of such a protein likely affects the DNA methylation pattern, which in turn could also affect the expression of specific genes similar to previous reports for *Synechocystis* (Gärtner et al., 2019). In this context, DNA methylation and its impact on nitrogen fixation were investigated in *Trichodesmium erythraeum* IMS101 (Walworth et al., 2017). However, in the latter cytosine methylation was not involved in the regulation of the nitrogen fixation (*nif*) genes directly. Nevertheless, the authors proposed a possible indirect effect on nitrogen fixation by influencing the expression of other nitrogen assimilatory genes. However, this is rather speculative and hence all these aspects need to be further investigated in PCC 7712 and PCC 7601. Meanwhile, there are also numerous hypothetical proteins defined uniquely in the PCC 7712 genome. Therefore, further comprehensive *in silico* analyses and physiological experiments are needed to understand the mechanism behind the difference in nitrogen fixation ability.

## Conclusion

Here, we evaluated the genome of *Tolypothrix* sp. PCC 7712 which was previously introduced as a novel biofilm-forming strain with high application potential for photobiotechnological applications. Thereby, we discovered its high similarity to the genome of *Tolypothrix* sp. PCC 7601 (often referred to as *Fremyella diplosiphon*). Despite these significant similarities at the genome level, PCC 7712 was able to reduce N_2_, while PCC 7601 neither shows nitrogenase activity, nor heterocyst formation. This is surprising, as the genome encodes for all necessary proteins currently known to be involved in nitrogen fixation. Thereby, these strains may become interesting models for research focusing on understanding the process of nitrogen fixation in cyanobacteria. It also shows, that a classification based on genome comparison only does not necessarily end up with close relatives also exhibiting the same physiological behavior.

## Data availability statement

The datasets presented in this study are available at the Genbank database under the following accession numbers: PRJNA625426 for PCC7712 and PRJNA625641 for PCC7601.

## Author contributions

MB: data curation, experiment, and writing—original draft preparation. MB, DP, RK, UR, SK, and KB: methodology and writing—review and editing. KB and SK: conceptualization, methodology, formal analysis, supervision, and writing—review and editing. All authors contributed to the article and approved the submitted version.

## Conflict of interest

The authors declare that the research was conducted in the absence of any commercial or financial relationships that could be construed as a potential conflict of interest.

## Publisher’s note

All claims expressed in this article are solely those of the authors and do not necessarily represent those of their affiliated organizations, or those of the publisher, the editors and the reviewers. Any product that may be evaluated in this article, or claim that may be made by its manufacturer, is not guaranteed or endorsed by the publisher.

## Footnotes

^1^ https://sourceforge.net/projects/bbmap/

^2^ https://github.com/rrwick/Porechop

^3^ https://github.com/nanoporetech/medaka

^4^ https://www.ncbi.nlm.nih.gov/genome/annotation_prok/

^5^ https://usegalaxy.eu/
