# Supplementary material for: Whole-genome sequence of the filamentous diazotrophic cyanobacterium Tolypothrix sp. PCC 7712 and its comparison with non-diazotrophic Tolypothrix sp. PCC 7601
Source: Front Microbiol. 2022 Nov 8;13:1042437. doi: 10.3389/fmicb.2022.1042437 (PMC9679502; doi:10.3389/fmicb.2022.1042437)
Supplement: Supplementary file 2 [file Data_Sheet_1.pdf]

Supplementary Material

1 Supplementary Data

Table S1. (a) Sequencing raw read statistics of Illumina and Nanopore data for both cyanobacteria (b) Assembly statistics using i) only Illumina reads, ii) Illumina and Nanopore reads in hybrid assembly mode of unicycler, and iii) only Nanopore reads and subsequent polishing with Nanopore and Illumina reads iv) Nanopore and two Illumina sequencing reads in hybrid assembly mode of unicycler.

a)

	**PCC 7601**		**PCC 7712**		
	Illumina	*Nanopore*	*Illumina-1*	*Illumina-2*	*Nanopore*
**Total output (Gb)**	1.0	0.4	1.3	1.0	1.6
**Coverage**	100x	40x	130x	100x	160x
**Number of reads**	6,799,374	38,591	6,585,256	5,319,808	348,080
**Mean quality (Q score)**	28.5	11.9	33.5	33.6	12.7
**Mean read length (bp)**	151	3,632	192	188	2,882
**Read length N50 (bp)**	-	27,765	-	-	7,971

b)

	**PCC 7601**			**PCC 7712**		
	i) Illumina only	*ii) Hybrid*	*iii) Long reads + polishing*	*i) Illumina only*	*ii) Hybrid*	*iv) Hybrid*
**Number of contigs**	669	35	16	885	36	16
**Largest contig (Gb)**	0.2	3.0	9.0	0.2	3.3	9.0
**Total length (Gb)**	9.9	10.2	10.1	9.9	10.2	10.1
**N50**	28 kb	2.7 Gb	9.0 Gb	20 kb	1.3 Gb	9.0 Gb
**Completeness**		99.11%	99.11%		99.11%	99.11%
**Contamination**		1.11%	0.98%		1.44%	0.00%

Table S2. NCBI PGAP annotation statistics.

	PCC 7712	PCC 7601
Genes (total)	8,111	8,309
CDSs (total)	7,990	8,187
Genes (coding)	7,728	7,831
CDSs (with protein)	7,728	7,831
Genes (RNA)	121	122
rRNAs	5, 5, 5 (5S, 16S, 23S)	5, 5, 5 (5S, 16S, 23S)
complete rRNAs	5, 5, 5 (5S, 16S, 23S)	5, 5, 5 (5S, 16S, 23S)
tRNAs	102	103
ncRNAs	4	4
Pseudo Genes (total)	262	356
CDSs (without protein)	262	356
Pseudo Genes (ambiguous residues)	0 of 262	0 of 356
Pseudo Genes (frameshifted)	51 of 262	137 of 356
Pseudo Genes (incomplete)	200 of 262	214 of 356
Pseudo Genes (internal stop)	35 of 262	44 of 356
Pseudo Genes (multiple problems)	23 of 262	38 of 356
CRISPR Arrays	16	17

Table S3. Accession numbers of selected strains for dRep Mash Clustering.

**Strain**	**Genbank accession**
Anabaena cylindrica PCC 7122	GCA_000317695.1
Calothrix brevissima NIES-22	GCA_002367995.1
Calothrix sp. PCC 7103	GCA_000331305.1
Calothrix sp. PCC 7716	GCA_019977735.1
Fischerella thermalis JSC-11	GCA_000231365.2
Leptolyngbya sp. BL0902	GCA_016403105.1
Leptolyngbya sp. PCC 7376	GCA_000316605.1
Microcystis sp. MC19	GCA_003019735.1
Microcystis aeruginosa NIES-88	GCA_001578085.1
Nostoc punctiforme PCC 73102	GCA_000020025.1
Nostoc sp. ATCC 53789	GCA_009873495.1
Nostoc sp. NIES-3756	GCA_001548375.1
Nostoc sp. PCC 7107	GCA_000316625.1
Nostoc sp. PCC 7120	GCA_000009705.1
Prochlorococcus marinus subsp. marinus str. CCMP1375	GCA_000007925.1
Synechococcus elongatus UTEX 2973	GCA_000817325.1
Synechocystis sp. PCC 6803	GCA_000009725.1
Tolypothrix bouteillei VB521301	GCA_000760695.4
Tolypothrix campylonemoides VB511288	GCA_000828075.3
Tolypothrix sp. PCC 7910	GCA_011769525.1
Tolypothrix tenuis PCC 7101	GCA_002368295.1

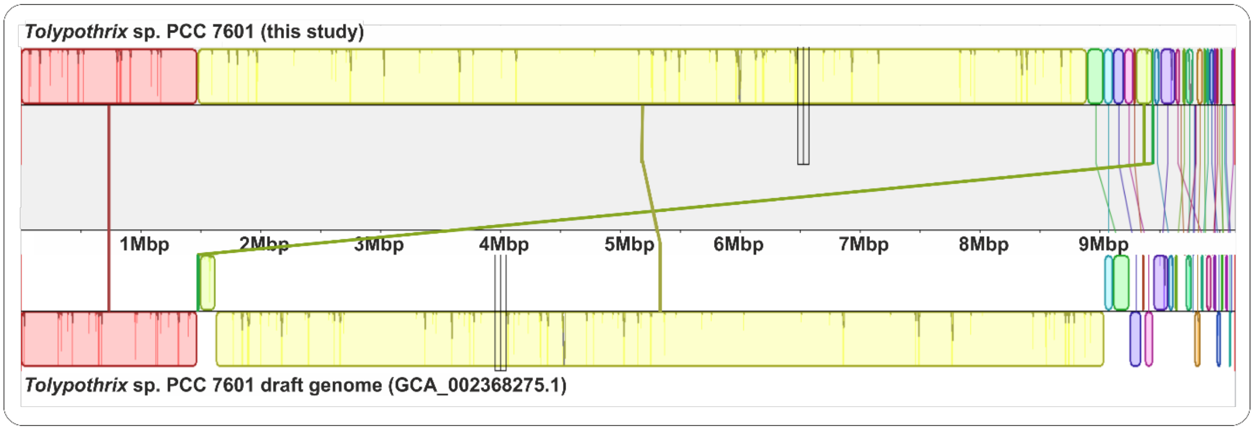

Figure S1. Whole-genome alignment of PCC 7601 sequenced in this study and the draft genome deposited in Genbank under the GCA_002368275.1 accession number.
